# Meetings of the Sections

**Published:** 1867-06

**Authors:** 


MEETINGS OF THE SECTIONS.

Dr. Murphy announced the places of meeting of the different sections of the Association, viz.:

*Chemistry and Materia Medica*—Medical College of Ohio.

*Medical Jurisprudence and Hygiene*—Medical College of Ohio.

*Psychology*—Dental College, College street, between Sixth and Seventh.

*Practical Medicine and Obstetrics*—Dental College.

*Surgery*—Medical College of Ohio.

*Meteorology, Medical Typography, and Epidemic Diseases*—Medical College of Ohio.

